# The Role of Patients’ Age on Their Preferences for Choosing Additional Blood Pressure-Lowering Drugs: A Discrete Choice Experiment in Patients with Diabetes

**DOI:** 10.1371/journal.pone.0139755

**Published:** 2015-10-07

**Authors:** Sieta T. de Vries, Folgerdiena M. de Vries, Thijs Dekker, Flora M. Haaijer-Ruskamp, Dick de Zeeuw, Adelita V. Ranchor, Petra Denig

**Affiliations:** 1 Department of Clinical Pharmacy and Pharmacology, University of Groningen, University Medical Center Groningen, Groningen, The Netherlands; 2 Institute for Transport Studies, University of Leeds, Leeds, United Kingdom; 3 Department of Health Psychology, University of Groningen, University Medical Center Groningen, Groningen, The Netherlands; Kermanshah University of Medical Sciences, ISLAMIC REPUBLIC OF IRAN

## Abstract

**Objectives:**

To assess whether patients’ willingness to add a blood pressure-lowering drug and the importance they attach to specific treatment characteristics differ among age groups in patients with type 2 diabetes.

**Materials and Methods:**

Patients being prescribed at least an oral glucose-lowering and a blood pressure-lowering drug completed a questionnaire including a discrete choice experiment. This experiment contained choice sets with hypothetical blood pressure-lowering drugs and a no additional drug alternative, which differed in their characteristics (i.e. effects and intake moments). Differences in willingness to add a drug were compared between patients <75 years (non-aged) and ≥75 years (aged) using Pearson χ^2^-tests. Multinomial logit models were used to assess and compare the importance attached to the characteristics.

**Results:**

Of the 161 patients who completed the questionnaire, 151 (72%) could be included in the analyses (mean age 68 years; 42% female). Aged patients were less willing to add a drug than non-aged patients (67% versus 84% respectively; P = 0.017). In both age groups, the effect on blood pressure was most important for choosing a drug, followed by the risk of adverse drug events and the risk of death. The effect on limitations due to stroke was only significant in the non-aged group. The effect on blood pressure was slightly more important in the non-aged than the aged group (P = 0.043).

**Conclusions:**

Aged patients appear less willing to add a preventive drug than non-aged patients. The importance attached to various treatment characteristics does not seem to differ much among age groups.

## Introduction

There is growing interest to tailor drug treatment to the individual patient's clinical and personal needs. In general, the incorporation of patient preferences in treatment decisions is high on the agenda in our society. Particularly in drug treatment it is important to take patient preferences into account since such preferences are related to a patient’s willingness to take a drug. The willingness to take a drug improves adherence and leads to a more effective treatment [1–3].

A patient’s willingness to take a drug is influenced by factors related to the drug, the physician, the disease, and patient’s own characteristics [4–7]. Drug-related factors include expected effects on, for example, life extension or factors related to quality of life, such as good health, ease of taking the drug and burden of adverse drug events (ADEs) [1]. Patients often value life extension as one of the most important drug effects [8–11]. On the other hand, many aged or frail patients are likely to value quality of life over life extension [12–14]. Therefore, one may expect that preferences for specific drugs and the willingness to add a treatment are influenced by a patient’s age or life-expectancy, as has been shown previously [12,15,16].

The role of patient’s age on drug choices may be particularly important for preventive treatment, such as the use of blood pressure-lowering drugs in patients with type 2 diabetes to reduce their risk for cardiovascular morbidity and mortality [17]. With respect to balancing life extension with quality of life, the need for blood pressure-lowering drugs may be less in aged patients since 1) evidence of long-term benefit in aged patients is lacking [18], 2) having a high blood pressure level is usually not perceived as burdensome [19], and 3) adverse events related to these drugs are common in the aged [20,21]. In general, patients with type 2 diabetes appear to have lower necessity beliefs for blood pressure-lowering drugs than, for instance, for glucose-lowering drugs [19,22]. Currently, little is known about preferences for choosing blood pressure-lowering treatment among aged and non-aged patients.

A useful and commonly used method to assess patient preferences for treatment is the discrete choice experiment [23,24]. In such experiments, a hypothetical situation is presented to the patient with treatment alternatives described by their characteristics, or so-called attributes [25]. The relative importance of the attributes can be inferred from the choices made [26]. This method is useful because people have to make trade-offs between positive and negative consequences of a choice, similar to decisions that they have to make in practice.

The aim of this study is to assess whether patients’ willingness to add a blood pressure-lowering drug and the importance they attach to specific treatment characteristics differ among age groups in patients with type 2 diabetes.

## Materials and Methods

### Study design and participants

This study was conducted in 2014 and has a cross-sectional design in which we compared patients <75 years with ≥75 years of age. Patients were eligible to participate when they were aged ≥18 years and had been prescribed at least an oral glucose-lowering and a blood pressure-lowering drug in the past 6 months. We aimed to include 150 patients in our study, since it has been shown that the precision of discrete choice experiments rapidly decreases at sample sizes less than 150 [26]. Pharmacists in the northern part of the Netherlands sent invitation letters to eligible patients identified from their electronic records. Patients who gave written informed consent received a questionnaire including general questions such as Cantril’s ladder to assess a patient’s quality of life [27], and subsequently the discrete choice experiment to evaluate their treatment preferences. Patients were called when they did not return the questionnaire within two months or in case of missing data in the general questions. In case of missing data in the discrete choice experiment, the questionnaire was returned to the patient for further completion. The Medical Ethics Committee of the University Medical Center Groningen (METc UMCG) in the Netherlands determined that ethical approval was not needed for this study (reference number M14.150721). The study was carried out in accordance with the Code of Ethics of the World Medication Association (Declaration of Helsinki) for experiments involving humans.

### Outcome variable

The outcome variable in this study was the choice patients made in a hypothetical situation with respect to adding a blood pressure-lowering drug.

### Discrete choice experiment

Attributes in the discrete choice experiment were based on a two-step literature review. In the first step, the literature was assessed for factors which may influence a patient’s willingness to take blood pressure-lowering drugs or drugs for primary cardiovascular disease prevention in general. This review revealed that lowering the blood pressure, achieving risk reduction of complications (myocardial infarction, stroke), reducing ADEs and improving quality of life were relevant factors [4–7]. During the second step, previous studies with discrete choice experiments to assess patient preferences for any drug were screened. This screening revealed two additional attributes, that is, costs of treatment and number of tablets needed per day [28–35]. We decided not to include costs in our experiment since expenditure for preventive drugs, such as blood pressure-lowering drugs, are covered by the health insurance in the Netherlands.

Levels of efficacy were established using clinical trial data of blood pressure-lowering treatment effects and cardiovascular risk reduction between tight and less tight blood pressure control, and using the UKPDS risk engine for assessing differences among different ages [36]. We estimated risks of complications or death within the next 5 years. Levels for ADEs were based on the prevalence of known ADEs for blood pressure-lowering drugs, such as cough and headache, as reported in the national drug compendium for healthcare professionals. Levels for the intake moments were based on possible schemes mentioned in the literature [37,38]. The list of attributes and levels was discussed and finalized by interviews with ten experts (2 nurse practitioners, 3 general practitioners, 2 specialists, and 3 pharmacists) (Table 1).

**Table 1:** Overview of the attributes and levels used in the discrete choice experiment.

Attributes	Levels	Coding
Blood pressure level^1^	Remains 160*	160
	Decrease from 160 to 140	140
	Decrease from 160 to 150^‡^	150
Risk of death by a heart attack or stroke in the next 5 years^1^	13 of the 100 die and 87 don’t*	0.13
	9 of the 100 die and 91 don’t	0.09
	11 of the 100 die and 89 don’t^‡^	0.11
Risk of limitations due to a heart attack, such as fatigue and difficulty walking in the next 5 years^1^	7 of the 100 get limitations and 93 don’t*	0.07
	5 of the 100 get limitations and 95 don’t	0.05
	6 of the 100 get limitations and 94 don’t^‡^	0.06
Risk of limitations due to a stroke, such a speech problems and forgetfulness in the next 5 years^1^	7 of the 100 get limitations and 93 don’t*	0.07
	5 of the 100 get limitations and 95 don’t	0.05
	6 of the 100 get limitations and 94 don’t ^‡^	0.06
Risk of side effect^3^, such as cough and headache^1^	No side effects*	0
	5 of the 100 get side effects and 95 don’t	0.05
	10 of the 100 get side effects and 90 don’t^‡^	0.10
Intake moment^2^	1 tablet in the morning*	-
	1 tablet in the morning and 1 in the evening^‡^	
	1 combination tablet	
	2 tablets in the morning^†^	

^1^ Continuous variable;

^2^ Categorical variable;

^3^ Side effect used as lay-term for adverse drug events.

* Level used for ‘no additional drug’ option (never used for the additional drug options).

^‡^ Levels used for the non-preferable drug in the dominant choice set.

^†^ Reference category in the categorical attribute.

A d-efficient design [26] of 30 choice sets, divided in three blocks, was generated using Ngene (version 1.1.1). No restrictions of level combinations were included. Patients were randomly assigned to one of the blocks containing ten choice sets. This blocking was used to reduce the cognitive burden of the patients. Patients had to imagine that they were using one blood pressure-lowering drug and that their blood pressure was uncontrolled (160 mmHg). In the choice sets, patients could indicate how they would want to continue their treatment, choosing from hypothetical treatment options presented as ‘no additional drug’, ‘additional drug A’, or ‘additional drug B’. The profile of ‘no additional drug’ described the situation as presented in the case, whereas the profiles of ‘additional drug A’ and ‘additional drug B’ were generated with the d-efficient design. Patients were asked to complete the ten choice sets plus a choice set in which one drug was preferable on all attributes compared to the other drug. This dominant choice set was added to identify the responders who may not understand the task.

**Fig 1:**
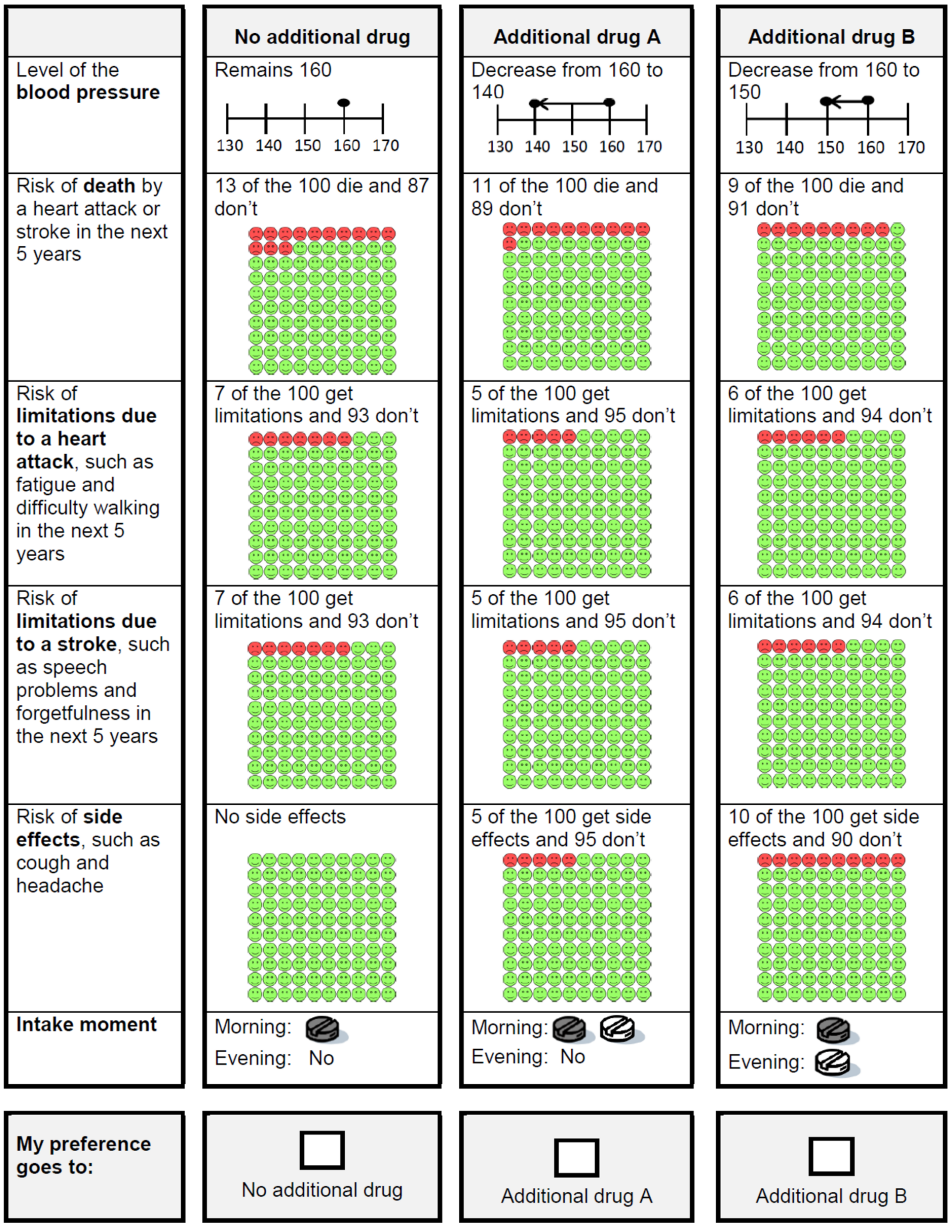
Example of a choice set presented in the questionnaire.

A pilot study was conducted in which ten patients with type 2 diabetes completed the full questionnaire. Based on this pilot study, minor changes in wordings were made and a separate instruction form for the choice sets was included. An example of a final choice set is presented in Fig 1.

### Patients’ age and life-expectancy

In the questionnaire, patients were asked their age. We used a cut-off level of ≥75 years to define aged patients. This cut-off level has been used in guidelines [39] and observational studies [40] looking at age differences for preventive treatment. To test its value in relation to perceived life-expectancy, we additionally asked patients an open-ended question: “In 2012, men became on average 75 years old and women on average 80 years old [41]. How old do you think you will become?”. With this we determined that age was a reasonable proxy for self-reported life-expectancy (S1 Table).

### Additional data collection

Pharmacists provided the age and gender for all eligible patients, as well as overviews of prescribed drugs in the last six months for those patients who gave written informed consent. The Anatomical Therapeutic Chemical (ATC) classification system of the World Health Organization was used to classify the prescribed drugs.

### Statistical analyses

Descriptive statistics are presented for patient characteristics. Differences in age and gender between patients who completed the study and those who did not participate or complete the study were assessed using the T-test and Pearson χ^2^-test respectively. Differences in characteristics between non-aged and aged patients were tested using Pearson χ^2^-tests for categorical variables and Mann-Whitney U tests for non-normally distributed, continuous variables. The Fisher freeman-halton test was used for categorical variables in which one or more cells contained less than five patients.

Differences in the number of patients who chose at least once an additional drug on the choice sets (willing to add) versus never (unwilling to add) were compared between the age groups using the Pearson χ^2^-test. The choices were further analysed using multinomial logit models (asclogit function in Stata) to assess 1) the willingness to add a blood pressure-lowering drug when controlling for all attributes, and 2) the relative importance of the attributes. Four models were assessed, that is, one including all patients, one including non-aged patients, one including aged patients, and one with all patients in which the interaction terms between these two age groups and the attributes were included.

We followed the random utility model by assuming that patients choose the alternative in each choice set which maximizes their utility. The estimated model was the following:

$$\begin{array}{l} U=\;V+\text{ ε }\\ \;=\beta 0\text{additional drug }+\beta 1\text{blood pressure}+\beta 2\text{death}+\beta 3\text{limitations heart attack}+\\ \;\beta 4\text{limitations stroke}+\beta 5\text{ADEs}+\beta 6\text{one in morning one in evening}+\\ \;\beta 7\text{combination tablet}+\varepsilon \end{array}$$

with *U* indicating the utility that a patient assigns to a treatment which is the sum of a systematic, explainable component *V* and a random, unexplainable component ε. The explainable component is a function of the attributes of the alternatives. The constant *β*0 indicates the relative weight patients place on choosing an additional blood pressure-lowering drug versus no additional blood pressure-lowering drug when controlling for the attributes. The *β*1 to *β*7 coefficients indicate the relative importance of each of the attributes. Coefficients reflect continuous variables of the attributes, except *β*6 and *β*7 which reflect the dummy-coding of respectively the level one drug in the morning and one in the evening (coded as 1) versus two drugs in the morning (coded as 0), and the level combination tablet (coded as 1) versus two drugs in the morning (coded as 0). The sign of the beta-coefficients indicates whether the effect on the utility is positive or negative. Attributes with beta-coefficients with a two-sided P-value <0.05 were considered as being important for the treatment choice. Important attributes were ranked according to their relative importance. This was determined by calculating the difference between the smallest part worth utility and the largest part-worth utility of the levels of an attribute, and dividing this difference by the sum of the difference scores for all attributes [42].

Patients who chose the non-preferable drug in the dominant choice set were excluded from the analyses. Sensitivity analyses were conducted in which these patients were not excluded. Additional sensitivity analyses were conducted to assess the multinomial logit model for patients <65 years and patients aged ≥80 years. The analyses were conducted using Stata version 13 (Stata Corp., College Station, TX).

## Results

**Fig 2:**
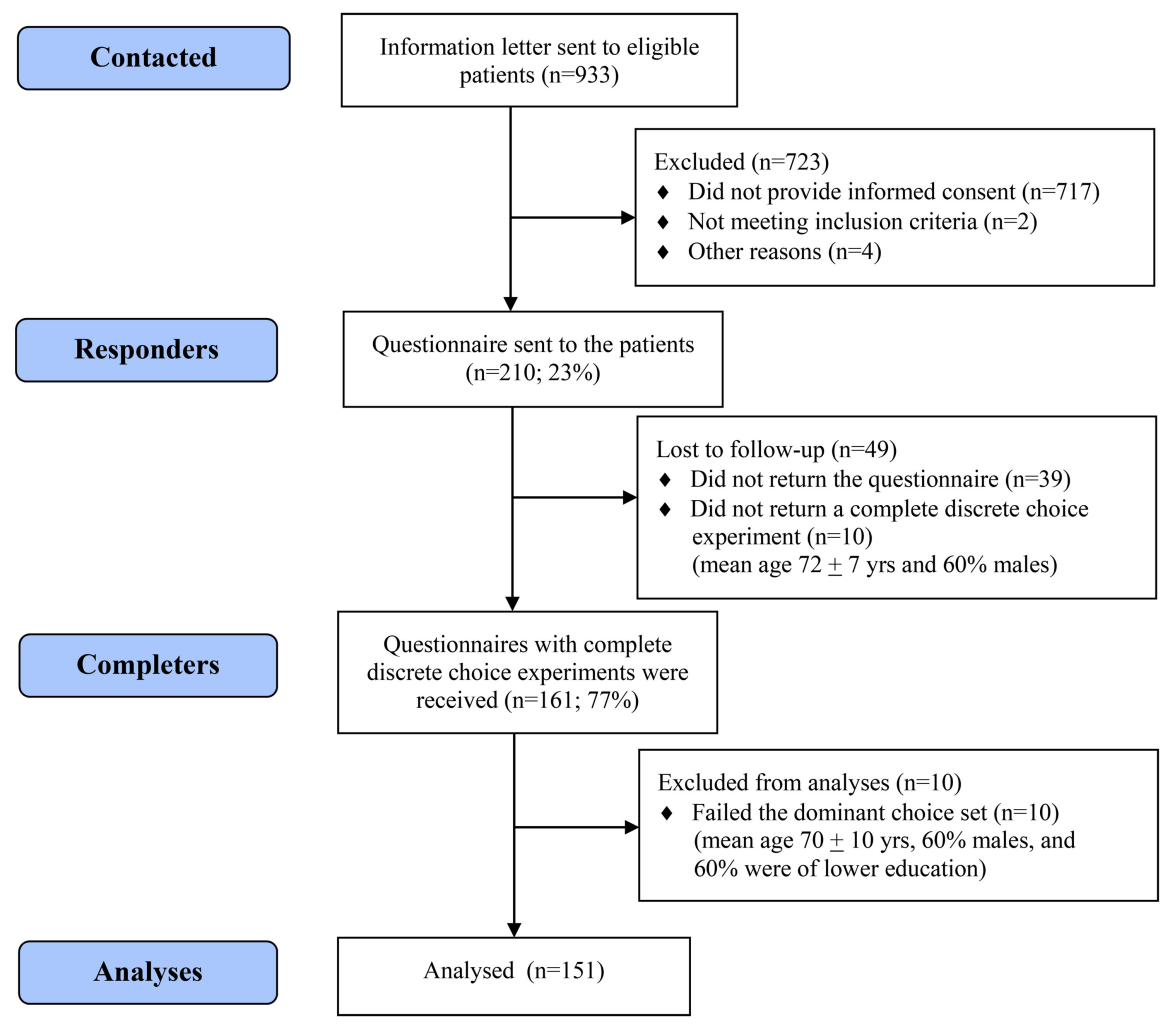
Patient inclusion flow-chart.

Three pharmacies sent information letters to 933 patients, resulting in 210 eligible consenting patients. Of these, 161 completed the questionnaire (Fig 2). There was no significant age difference between these completers and the non-completers (69 and 69 years, P = 0.310), but less females completed the study (42% versus 52% females, P = 0.025). This difference became insignificant in an additional analysis per age group (data not shown). In the final analyses, 151 were included. The included patients were on average 68 ± 9 yrs and the majority were males (58%). Metformin was the most commonly prescribed glucose-lowering drug in both aged and non-aged patients (Table 2). Agents acting on the renin-angiotensin system (RAS-inhibitors) were the most commonly prescribed blood pressure-lowering drug class in non-aged patients, whereas the *β*-blockers were the most commonly prescribed class in aged patients. Most of the patients reported that their blood pressure was <160 mmHg, that they used one or two drugs to lower their blood pressure, and that they preferred to leave decisions about their drugs to the general practitioner (Table 2).

**Table 2:** Patient characteristics per age group.

Characteristic	All	<75 years	≥75 years	P-value
Included patients	151	106	45	
Mean age (SD)	68 (9.2)	64 (7.1)	79 (3.6)	
Females (%)	64 (42.4)	40 (37.7)	24 (53.3)	0.076^1^
Median body mass index (IQR)	28 (26–32)	29 (27–33)	26 (24–29)	0.000^2^
Education (%)				0.472^3^
Lower education^a^	86 (57.0)	59 (55.7)	27 (60.0)	
Middle education^b^	44 (29.1)	34 (32.1)	10 (22.2)	
Higher education^c^	17 (11.3)	11 (10.4)	6 (13.3)	
Other	4 (2.7)	2 (1.9)	2 (4.4)	
Smoking				0.011^1^
Current smokers	22 (14.6)	16 (15.1)	6 (13.3)	
Past smokers	75 (49.7)	60 (56.6)	15 (33.3)	
Non smokers	54 (35.8)	30 (28.3)	24 (53.3)	
Median quality of life (IQR)◦	3 (3–5)	3 (3–4)	4 (3–5)	0.351^2^
Classes of prescribed blood pressure-lowering drugs (ATC code)*				
Centrally acting antihypertensives (C02)	2 (1.3)	0 (0.0)	2 (4.4)	0.089^3^
Diuretics (C03)	49 (32.7)	31 (29.5)	18 (40.0)	0.210^1^
*β*-Blockers (C07)	87 (58.0)	56 (53.3)	31 (68.9)	0.077^1^
Calcium channel blockers (C08)	33 (22.0)	20 (19.1)	13 (28.9)	0.182^1^
Agents acting on the renin-angiotensin system (C09)	104 (69.3)	74 (70.5)	30 (66.7)	0.643^1^
Combination tablet^‡^	27 (18.0)	16 (15.2)	11 (24.4)	0.179^1^
Classes of prescribed glucose-lowering drugs (ATC code)*				
Insulin (A10A)	33 (22.0)	27 (25.7)	6 (13.3)	0.093^1^
Biguanides (metformin) (A10BA)	135 (90.0)	96 (91.4)	39 (86.7)	0.373^1^
Sulfonamides (A10BB)	58 (38.7)	39 (37.1)	19 (42.2)	0.558^1^
Combination Metformin and Sulfonamide (A10BD02)	1 (0.7)	1 (1.0)	0 (0.0)	1.000^3^
Thiazolidinediones (A10BG)	1 (0.7)	1 (0.95)	0 (0.0)	1.000^3^
Dipeptidyl peptidase 4 inhibitors (A10BH)	13 (8.7)	9 (8.6)	4 (8.9)	1.000^3^
Liraglutide (A10BX07)	2 (1.3)	2 (1.9)	0 (0.0)	1.000^3^
Use of lipid-lowering drugs (%)*				0.019^3^
No lipid-lowering drug	27 (18.0)	13 (12.4)	14 (31.1)	
1 lipid-lowering drug	117 (78.0)	88 (83.8)	29 (64.4)	
2 lipid-lowering drugs	6 (4.0)	4 (3.8)	2 (4.4)	
Drug burden expressed as median number of chronic treatments from 8 anatomical chapters (IQR)^ø^	3 (3–4)	3 (3–4)	4 (3–4)	0.025^2^
*High blood pressure*				
How serious do you think that having a high blood pressure is in general? (%)				0.584^3^
Very serious	20 (13.4)	13 (12.4)	7 (15.9)	
Reasonable serious	93 (62.4)	69 (65.7)	24 (54.6)	
A little serious	28 (18.8)	18 (17.1)	10 (22.7)	
Not serious	8 (5.4)	5 (4.8)	3 (6.8)	
How high was your systolic blood pressure during the last measurement conducted by your general practitioner or nurse practitioner? (%)				0.162^1^
<120 mmHg	10 (6.6)	7 (6.6)	3 (6.7)	
120–139 mmHg	66 (43.7)	52 (49.1)	14 (31.1)	
140–159 mmHg	47 (31.1)	32 (30.2)	15 (33.3)	
≥160 mmHg	16 (10.6)	9 (8.5)	7 (15.6)	
I do not know	12 (8.0)	6 (5.7)	6 (13.3)	
Number of patients who report ever having experienced a symptom of high blood pressure (%)	36 (23.8)	25 (23.6)	11 (24.4)	0.910^1^
*Blood pressure-lowering drugs*				
Number of drugs that the patients report to use for high blood pressure				0.102^1^
None	6 (4.0)	6 (5.7)	0 (0.0)	
One	79 (52.3)	55 (51.9)	24 (53.3)	
Two	38 (25.2)	29 (27.4)	9 (20.0)	
More than two	19 (12.6)	9 (8.5)	10 (22.2)	
I do not know	9 (6.0)	7 (6.6)	2 (4.4)	
Have you ever experienced a side effect of a blood pressure-lowering drug				0.511^1^
No	110 (72.9)	78 (73.6)	32 (71.1)	
Yes	24 (15.9)	18 (17.0)	6 (13.3)	
I do not know	17 (11.3)	10 (9.4)	7 (15.6)	
I prefer to leave decisions about my drugs to my general practitioner^†^	142 (94.0)	100 (94.3)	42 (93.3)	0.811^1^

^a^ No education; Elementary school; Junior secondary vocational education.

^b^ Junior general secondary education; Senior secondary vocational education.

^c^ Senior general secondary education; Higher professional education; University education.

SD = Standard deviation; IQR = Interquartile range; ATC = Anatomical Therapeutic Chemical.

^◦^ Measured with Cantril’s ladder [27] with a range of 1 (best)– 10 (worst possible life).

* N = 150 (medication overview of one patient was not extracted at the time of data collection since the patient had not given written informed consent yet).

^‡^ ATC codes: C03EA01, C07BB02, C09BA03, C09BA04, C09BA06, C09BB04, C09DA01, C09DA03, C09DA04, C09DA06, C09DB02.

^ø^ Drug burden was counted at the anatomical ATC level for the chapters: A, B, C, H, L, M, N, R (maximum of 8).

^†^ Statement adapted from [43]. Scored on a 6-point Likert scale and divided by (partially, totally) agree and (partially, totally) disagree. Number is presented for those who agree.

^1^ Pearson χ^2^-test;

^2^ Mann-Whitney U test;

^3^ Fisher freeman-halton test.

### Influence of age on willingness to add a blood pressure-lowering drug

Of the aged patients, 67% chose an additional drug in at least one of the ten presented choice sets. This percentage was significantly lower than in non-aged patients (84%; P = 0.017). The same is reflected in the drug choice model where the constant is more negative in the aged than in the non-aged (Table 3).

**Table 3:** Preferences of all patients and divided in patients aged <75 years (non-aged) and ≥75 years (aged).

Constant and attributes	All patients^a^			<75 years^b^			≥75 years^c^			P-value of the interaction between age groups and preferences^d^
	*Coefficient (95% CI)*	*P-value*	*Relative importance (ranking)* *	*Coefficient (95% CI)*	*P-value*	*Relative importance (ranking)* *	*Coefficient (95% CI)*	*P-value*	*Relative importance (ranking)* *	
Constant (additional drug)	-1.26 (-1.72 –-0.80)	0.000		-1.05 (-1.60 –-0.50)	0.000		-1.65 (-2.52 –-0.77)	0.000		0.257
Blood pressure	-0.08 (-0.10 –-0.07)	0.000	36.10 (1)	-0.09 (-0.11 –-0.08)	0.000	37.22 (1)	-0.06 (-0.09 –-0.03)	0.000	36.84 (1)	0.043
Death within the next 5 years	-22.13 (-28.75 –-15.51)	0.000	19.97 (3)	-21.79 (-29.59 –-13.99)	0.000	18.03 (3)	-24.43 (-37.32 –-11.54)	0.000	30.00 (3)	0.731
Limitations heart attack	-9.16 (-22.29–3.98)	0.172		-9.13 (-24.61–6.36)	0.248		-11.31 (-36.83–14.22)	0.385		0.886
Limitations stroke	-26.65 (-39.89 –-13.41)	0.000	12.03 (4)	-30.22 (-45.83 –-14.61)	0.000	12.50 (4)	-15.71 (-41.42–10.00)	0.231		0.344
Adverse drug events	-14.14 (-16.89 –-11.39)	0.000	31.90 (2)	-15.59 (-18.86 –-12.31)	0.000	32.24 (2)	-10.80 (-16.02 –-5.58)	0.000	33.16 (2)	0.128
Additional tablet in the evening	0.07 (-0.10–0.25)	0.424		0.13 (-0.08–0.34)	0.216		-0.10 (-0.44–0.24)	0.578		0.264
Combination tablet	0.13 (-0.05–0.31)	0.151		0.10 (-0.11–0.31)	0.361		0.21 (-0.12–0.54)	0.206		0.566

^a^ Number of observations 4,530 (151 patients * 10 choice sets * 3 alternatives per choice set).

^b^ Number of observations 3,180 (106 patients * 10 choice sets * 3 alternatives per choice set).

^c^ Number of observations 1,350 (45 patients * 10 choice sets * 3 alternatives per choice set).

^d^ Interaction between preferences and age groups added to the model of all patients.

* Determined by calculating the difference between the smallest part worth utility and the largest part-worth utility of the levels of an attribute, and dividing this difference by the sum of the difference scores for all attributes [42].

CI = Confidence interval.

### Influence of age on importance attached to drug attributes

Drug attributes that significantly influenced the choices for an additional blood pressure-lowering drug in the total group were the effect on the blood pressure, the risk of experiencing ADEs, the risk of death within the next 5 years, and the risk on limitations due to stroke within the next 5 years (Table 3). These results were similar in the analyses including only non-aged patients. The risk of limitations due to a stroke within the next 5 years did not significantly contribute to the choice of an additional drug in the aged group. Ranking of the relative importance of the attributes revealed that in both aged and non-aged patients, the effect on the blood pressure was the most influencing attribute, followed by the risk of experiencing ADEs and the risk of death within the next 5 years (Table 3).

A sensitivity analysis in which the patients who failed the dominant choice set were included, revealed similar results (S2 Table). In addition, sensitivity analyses including only younger patients (<65 years) or older patients (≥80 years) showed the same direction for all coefficients but some coefficients became insignificant (S3 Table).

Only the impact of the blood pressure-lowering effect turned out to be significantly different among the age groups (Table 3, interaction, P = 0.043). This finding indicates that the effect of an additional drug on the blood pressure level was seen as more important by non-aged patients than aged patients.

## Discussion

This study in patients with type 2 diabetes shows that aged patients were less willing to add a blood pressure-lowering drug than non-aged patients when they had to imagine that their blood pressure was too high. The effect of a drug on the blood pressure was more important for non-aged patients than aged patients. The effects on the risk of death within the next 5 years and experiencing ADEs were important drug characteristics for choosing a drug in both age groups. For non-aged patients, also the risk of limitations due to a stroke was important.

Previously, it was found that patients with a limited life-expectancy have a decreased willingness to add treatment because they may value quality of life over life extension [12,15]. On the other hand, it was also found that a large proportion of such patients remain willing to undergo burdensome treatment for a small risk reduction of death [12]. Our study confirms the finding that aged patients are less willing to add a drug than non-aged patients. The finding that the drug effect on reducing the risk of death within the next 5 years was of similar importance for aged and non-aged patients may be surprising, but fits with the finding that many aged patients remain willing to undergo burdensome treatment for a risk reduction of death [12]. This might be explained by the fact that the aged group still perceived they had sufficient life-expectance.

Previous studies have not directly compared the importance attached to various treatment outcomes between aged and non-aged patients. Our study revealed that most characteristics were similarly valued by aged patients in comparison to non-aged patients. Both groups attached a similar importance, for example, to the risk of ADEs, and in both groups was the effect on the blood pressure ranked as most important attribute. However, the effect on the blood pressure was the only attribute that significantly differed in importance between aged and non-aged patients. This finding could imply that aged patients are less willing to add a blood pressure-lowering drug because they believe that decreasing the blood pressure is of less importance. Whether this is influenced by the current advise of guidelines, and thus practitioners, to take a patient’s age or life-expectancy into account in setting blood pressure targets [44,45], remains to be determined.

In both age groups, the choice for a blood pressure-lowering drug was not significantly influenced by the risk reductions in limitations in daily life due to a heart attack. This finding may be due to a more subjective interpretation of such an attribute compared to an attribute such as death [46]. However, the preferences of the non-aged patients were influenced by a similar attribute, that is, the risk of limitations due to a stroke. Therefore, it seems that reducing potential limitations due to a heart attack from 7% to 5% was not decisive for the patients' treatment choices, whereas a similar reduction for limitations due to stroke was. These findings are comparable with a previous study showing that patients are less willing to risk cognitive disability than physical disability [12]. For the aged patients, however, reducing potential limitations due to a stroke from 7% to 5% was not critical. Possibly, the presented problems, i.e. speech problems and forgetfulness, may have been perceived by aged patients as an expected part of aging [47]. Presenting more severe problems, such as becoming dependent on others, might have increased the relevance of this aspect since maintaining independent is important for aged patients [48,49].

In our study, patients’ preferences for a blood pressure-lowering drug were not significantly influenced by the intake moment of the drug. A previous discrete choice experiment in patients with type 2 diabetes showed that the intake moment influences patient preferences, but that this is more important for patients who are taking less than five drugs a day compared to patients with five or more drugs per day [50]. This may explain the non-significant influence of the intake moment in our study since many patients were prescribed not only glucose-lowering and blood pressure-lowering drugs but also lipid-lowering drugs and drugs for other chronic diseases.

This study was conducted in the north of the Netherlands, which includes a mostly caucasian population. Selection bias may have occurred since only 17% of the contacted patients completed the questionnaire and less females completed the study. Regarding age there were no differences between those who completed the study and those who did not. Although we reached a sample size of 150 patients, it should be noted that the included number of patients per age group was lower which may have reduced the efficiency of the models for the separate groups. This limitation may especially apply to the aged group (≥75 years) since only 45 patients were included. In the aged group, more blood pressure-lowering drugs and especially *β*-blockers were used than in the non-aged group. However, a post-hoc analysis showed that the reported use of two or more blood pressure-lowering drugs was not associated with less willingness to add a drug (data not shown). In this study, age seemed to be a reasonable proxy for life-expectancy. There are also some strengths and limitations to a discrete choice experiment. A major strength is that it comes close to the trade-offs and choices that have to be made in real life, and can thus provide better estimates of the relative importance of different treatment characteristics than when asking patients to rate the importance of each characteristic separately. The evaluation of several treatment alternatives and attributes at one time reveals a rich source of data [26,51]. Moreover, the ‘no additional drug’ option was included which represents actual choices in practice. However, the choices that have to be made may be complex and may require high cognitive efforts [52]. Aged patients may have more problems with such choices than non-aged patients due to increased cognitive impairment. However, an exploratory study among older patients showed that cognitive impairment had no significant impact on the consistency of responses in a discrete choice experiment [53]. Furthermore, hypothetical situations have to be assessed in discrete choice experiments which do not necessarily represent actual behaviour [54,55]. Some patients may have difficulty in making hypothetical choices. To reduce complexity and effort, patients were presented a sample of ten choice sets and a pilot study in eligible patients was conducted to detect and resolve any difficulties. The pilot study revealed only a need for minor changes in wording and the use of a separate instruction card, and indicated that the task was doable for (aged) patients with type 2 diabetes. In addition, we excluded 10 patients from the analyses who failed the dominant choice set. There is discussion in the literature about the fairness of excluding such responders [56], but the sensitivity analysis in which these patients were included revealed similar results. A final limitation may be that for most of the attributes only a reduction in the frequency of a risk was presented. Other information may be relevant for patients. For instance, patients may want information about the severity and duration of the ADEs.

In conclusion, it is important to acknowledge that aged patients may be less willing to add a blood pressure-lowering drug than non-aged patients. This finding underlines the importance of discussing patients' preferences even when they prefer to leave the final treatment decision to their general practitioner. When choosing a drug, aged patients attach as much importance to reduce their risk of death within the next 5 years and of experiencing ADEs as non-aged patients. Therefore, treatment decisions in clinical practice should focus on quality of life as well as life extension in both age groups in which the individual patient’s preferences and willingness to add a drug should be taken into account.

## Supporting Information

S1 Table Association between self-reported life-expectancy and age. (DOCX)

S2 Table Preferences of patients aged <75 years and ≥75 years including patients who failed the dominant choice set. (DOCX)

S3 Table Preferences of patients aged <65 years and ≥80 years. (DOCX)

S1 Minimal Anonymized Dataset (DTA)
