# Supplementary material for: The Role of Patients’ Age on Their Preferences for Choosing Additional Blood Pressure-Lowering Drugs: A Discrete Choice Experiment in Patients with Diabetes
Source: PLoS One. 2015 Oct 7;10(10):e0139755. doi: 10.1371/journal.pone.0139755 (PMC4596700; doi:10.1371/journal.pone.0139755)
Supplement: S1 Table — (DOCX) [file pone.0139755.s001.docx]

**S1 Table. Association between self-reported life-expectancy and age^†^.**

|  |  | **Self-reported life-expectancy^*‡^** | | | |
| --- | --- | --- | --- | --- | --- |
|  |  | *≤2 years* | *>2 and ≤5 years* | *>5 and ≤10 years* | *>10 years* |
| **Age** | *<65 years* | 0 (0%) | 0 (0%) | 2 (4%) | 45 (96%) |
|  | *≥65 and <75 years* | 0 (0%) | 1 (2%) | 12 (24%) | 36 (73%) |
|  | *≥75 and <85 years* | 2 (8%) | 12 (48%) | 6 (24%) | 5 (20%) |
|  | *≥85 years* | 0 (0%) | 3 (75%) | 0 (0%) | 1 (25%) |

^*^ N = 125 since 26 patients did not report their life-expectancy.

^‡^ The age of how old patients expect they will become ranged from 70 to 100 for both males and females. Most patients reported to become 80 years (30% of the males, 37% of the females).

^†^ Fisher freeman-halton test revealed a P-value of <0.001.
