# Supplementary material for: The Role of Patients’ Age on Their Preferences for Choosing Additional Blood Pressure-Lowering Drugs: A Discrete Choice Experiment in Patients with Diabetes
Source: PLoS One. 2015 Oct 7;10(10):e0139755. doi: 10.1371/journal.pone.0139755 (PMC4596700; doi:10.1371/journal.pone.0139755)
Supplement: S2 Table — (DOCX) [file pone.0139755.s002.docx]

**S2 Table. Preferences of patients aged <75 years and ≥75 years including patients who failed the dominant choice set.**

| **Constant and attributes** | **<75 years^a^** | | | **≥75 years^b^** | | |
| --- | --- | --- | --- | --- | --- | --- |
|  | *Coefficient (95% CI)* | *P-value* | *Relative importance (ranking)** | *Coefficient (95% CI)* | *P-value* | *Relative importance (ranking)** |
| Constant (additional drug) | -0.79 (-1.31 – -0.26) | **0.003** |  | -1.19 (-1.98 – -0.39) | **0.003** |  |
| Blood pressure | -0.09 (-0.10 – -0.07) | **0.000** | 38.63 (1) | -0.05 (-0.07 – -0.03) | **0.000** | 35.52 (1) |
| Death within the next 5 years | -20.67 (-28.18 – -13.16) | **0.000** | 17.74 (3) | -21.58 (-33.22 – -9.94) | **0.000** | 30.66 (3) |
| Limitations heart attack | -6.24 (-21.15 – 8.66) | 0.412 |  | -8.55 (-31.67 – 14.57) | 0.469 |  |
| Limitations stroke | -25.55 (-40.55 – -10.56) | **0.001** | 10.97 (4) | -10.12 (-33.26 – 13.03) | 0.392 |  |
| Adverse drug events | -15.22 (-18.36 – -12.07) | **0.000** | 32.66 (2) | -9.52 (-14.22 – -4.82) | **0.000** | 33.82 (2) |
| Additional tablet in the evening | 0.13 (-0.07 – 0.33) | 0.193 |  | 0.04 (-0.26 – 0.35) | 0.778 |  |
| Combination tablet | 0.13 (-0.08 – 0.33) | 0.224 |  | 0.16 (-0.14 – 0.46) | 0.302 |  |

^a^ Number of observations 3,330 (111 patients * 10 choice sets * 3 alternatives per choice set).

^b^ Number of observations 1,500 (50 patients * 10 choice sets * 3 alternatives per choice set).

* Determined by calculating the difference between the smallest part worth utility and the largest part-worth utility of the levels of an attribute, and dividing this difference by the sum of the difference scores for all attributes [42].

CI = Confidence interval.
