# Supplementary material for: The Role of Patients’ Age on Their Preferences for Choosing Additional Blood Pressure-Lowering Drugs: A Discrete Choice Experiment in Patients with Diabetes
Source: PLoS One. 2015 Oct 7;10(10):e0139755. doi: 10.1371/journal.pone.0139755 (PMC4596700; doi:10.1371/journal.pone.0139755)
Supplement: S3 Table — (DOCX) [file pone.0139755.s003.docx]

**S3 Table. Preferences of patients aged <65 years and ≥80 years**.

| **Constant and attributes** | **<65 years^a^** | | | **≥80 years^b^** | | |
| --- | --- | --- | --- | --- | --- | --- |
|  | *Coefficient (95% CI)* | *P-value* | *Relative importance (ranking)** | *Coefficient (95% CI)* | *P-value* | *Relative importance (ranking)** |
| Constant (additional drug) | -0.09 (-0.83 – 0.65) | 0.813 |  | -0.24 (-1.82 – 1.34) | 0.765 |  |
| Blood pressure | -0.07 (-0.09 – -0.04) | **0.000** | 29.81 (2) | -0.06 (-0.11 – -0.01) | **0.017** | 35.71 (2) |
| Death within the next 5 years | -22.41 (-33.25 – -11.58) | **0.000** | 19.09 (3) | -15.81 (-40.24 – 8.62) | 0.205 |  |
| Limitations heart attack | -10.76 (-32.07 – 10.55) | 0.323 |  | 29.90 (-18.91 – 78.72) | 0.230 |  |
| Limitations stroke | -27.92 (-49.29 – -6.54) | **0.010** | 11.89 (4) | -20.15 (-69.44 – 29.15) | 0.423 |  |
| Adverse drug events | -18.42 (-22.99 – -13.86) | **0.000** | 39.22 (1) | -21.60 (-32.02 – -11.19) | **0.000** | 64.29 (1) |
| Additional tablet in the evening | 0.09 (-0.19 – 0.38) | 0.532 |  | -0.40 (-1.05 – 0.24) | 0.219 |  |
| Combination tablet | 0.07 (-0.22 – 0.36) | 0.645 |  | 0.07 (-0.54 – 0.68) | 0.828 |  |

^a^ Number of observations 1,560 (52 patients * 10 choice sets * 3 alternatives per choice set).

^b^ Number of observations 450 (15 patients * 10 choice sets * 3 alternatives per choice set).

* Determined by calculating the difference between the smallest part worth utility and the largest part-worth utility of the levels of an attribute, and dividing this difference by the sum of the difference scores for all attributes [42].

CI = Confidence interval.
